# Dietary Factors in Relation to *Helicobacter pylori* Infection

**DOI:** 10.1155/2014/826910

**Published:** 2014-12-02

**Authors:** Seyyed Ali Mard, Hossein Khadem Haghighian, Vahid Sebghatulahi, Bijan Ahmadi

**Affiliations:** ^1^Physiology Research Center (PRC) and Department of Physiology, Research Institute for Infectious Diseases of Digestive System, School of Medicine, Ahvaz Jundishapur University of Medical Sciences, Ahvaz 61357-15794, Iran; ^2^Department of Nutrition, Research Institute for Infectious Diseases of Digestive System, School of Paramedical, Ahvaz Jundishapur University of Medical Sciences, Ahvaz 61357-15794, Iran; ^3^Research Institute for Infectious Diseases of Digestive System, School of Medicine, Ahvaz Jundishapur University of Medical Sciences, Ahvaz 61357-15794, Iran; ^4^Research Institute for Infectious Diseases of Digestive System, School of Medicine, Kerman University of Medical Sciences, Kerman 7618747653, Iran

## Abstract

*Background and Aim. Helicobacter pylori* (HP) and diet are both risk factors for gastric cancer. The aim of this study was to evaluate the *Helicobacter pylori* infection and dietary habits common in Khuzestan province. *Methods.* This cross-sectional study was conducted in 2011–2013 on 374 patients. Participants were interviewed using a food frequency questionnaire and tissue sample of the antrum was sent for pathology lab. The histopathological major variables were graded on a scale of 3 (mild, moderate, and severe) and data analyzed using nonparametric tests. *Results.* In this study, of 160 patients (43%) that were determined, 8.1 percent had severe contamination. Among dietary patterns, relationship between energy intake and carbohydrate with *H. pylori* was significant. A direct association was found between mean daily intakes of sausage (*P* = 0.001) and burgers (*P* < 0.05) with HP infection. Low intake of fresh vegetables and fruits was the most significant risk factors (*P* < 0.05). *Conclusion.* There is a possibility that some dietary factors such as consumption of fast foods and low intake of fresh vegetables may increase the chance of HP and severity of this infection.

## 1. Introduction

*Helicobacter pylori* is a spiral, gram negative, acid tolerant, microaerophilic bacterium that lives in the stomach and duodenum [1, 2]. In Iran,* H. pylori* infection is present in nearly 90% of adult population [3] and appears to occur early in life, with >50% of children infected before age of 15 [4]. Despite the fact that the incidence of and mortality from gastric cancer have declined markedly worldwide over the past decades, gastric cancer is still the second most common cause of cancer-related death in the world [5]. Epidemiological data suggest that environmental factors are the predominant cause of this disease. The most important factors thought to be responsible for GC development are diet and* Helicobacter pylori* infection [6].

Besides the fact that* H. pylori* was introduced as a class I carcinogen [7], the infection is difficult to cure and requires various combination therapies [8]. Previous epidemiological studies have suggested that not only* H. pylori* infection but also varieties of environmental factors are important risk factors for GC [9–12]. It is believed that dietary factors may contribute to the* H. pylori* infection [13]. Adequate nutritional status, especially high consumption of fruits, vegetables, and vitamins, appears to protect against the pathological consequences of* H. pylori* infection [14]. Knoops et al. stressed the role of vitamin C as a chemopreventive factor in* H. pylori* gastric disorders [15, 16].

Furthermore, which environmental factor is involved in the development of GC among persons infected with* H. pylori* has rarely been investigated [17]. Therefore, we also investigated this point among* H. pylori*-infected subjects. The aim of this study was to evaluate whether there are differences in dietary habits between patients who were infected and subjects who were not infected.

## 2. Materials and Methods

The study was carried out in 2011–2013 in a group of patients from the Gastroenterological Unit of the Emam Khomeini Hospital in Ahvaz who had functional dyspepsia and had been referred for endoscopic examination of the upper digestive tract. To minimize any known confounding effects, the subjects with the following conditions have been excluded from the study: subjects with habits such as smoking, tobacco chewing, and alcohol consumption; on corticosteroid therapy at pharmacological levels for duration of more than 6 months; diabetes mellitus (except for easily controlled, non-insulin-dependent diabetes mellitus).

For all patients, energy and nutrient intakes were measured using a 3-day 24 h recall (two week days and one weekend day). Three-day food diary form contains foods at every meal, snack, and the amount consumed per person. A validated food frequency questionnaire [18] was also used to determine dietary habits during the previous year. The information was obtained through face to-face interviews, with standard food models, and a variety of measuring tools to evaluate intake. To help people to recall precise quantities of food eaten homemade dishes and cups were accounted. The participants were asked to indicate consumption of specified food in daily, weekly, monthly, and yearly bases. The selected frequency choice, given by the subjects for each food item from the food groups, was then converted to a weekly intake. All subjects were asked about the average frequency of intake and portion size of 125 items over the past year. The frequency of other food items was classified into 9 categories (never, 1–3/month, 1-2, 3-4, or 5-6/week, almost once/day, or 2-3, 4–6, or 7+ times/day).

Values of each food were converted to grams by using household measures. An interview on dietary habits was performed by a dietician. Nutrients were analyzed by Nutritionist IV software (N-Squared computing, Salem, OR, USA), which was modified for Iranian foods.

After upper GI endoscopy a tissue sample from antrum, body, and fundus in plate containing formalin buffer was sent to the pathology lab. All samples were examined by a pathologist. Contamination detection was performed with hematoxylin eosin (H&E). Semiquantitative method of scoring according to the Updated Sydney Classification System was undertaken. Statistical analysis was done using SPSS software (version 13). To correlate variables with normal and nonnormal distribution, Pearson's and Spearman's correlations, respectively, were used. All tests were two-tailed, and *P* < 0.05 was the significance threshold.

## 3. Result

In this study, 374 patients were evaluated, 182 patients (48%) of them were females and 192 (52%) were males. Based on pathological studies on* H. pylori* infection, 214 healthy subjects (57%) and 160 patients (43%) were diagnosed that 8% of them had severe contamination and 37.5% and 54.4% had moderate and mild contamination, respectively.

As it is shown in Table 1, there was a significant correlation between energy and carbohydrate intake with* H. pylori* infection. (*P* = 0.01, 0.02) There was also a negative and significant correlation between daily intake of fish (*P* = 0.001), olive oil (*P* = 0.002) and honey (*P* = 0.002), and peas and beans (*P* = 0.03) with* H. pylori* infection.

Table 2 shows that there was positive relation between sausages (*P* = 0.001), hamburgers (*P* = 0.002), fat mayonnaise (*P* = 0.002), and soft drinks (*P* = 0.001) with* H. pylori* infection.

Taking weekly tomatoes (*P* = 0.001), onions (*P* = 0.002), green pepper (*P* = 0.01), apple (*P* = 0.002), and citrus fruits (*P* = 0.001) was significantly lower than in healthy individuals (Table 3).

Among the micronutrients reported, there were significant differences in intake of vitamin C and folate between the healthy subjects and patient groups (Table 4).

Subjects with* H. pylori* infection were categorized into 3 groups: severe, moderate, and mild contamination (on Revised Sydney System).

As it is shown in Table 5, there were negative and significant correlation and significant difference in tomatoes, onions, green pepper, apple, citrus fruits, fish, olive oil, and honey and peas intake with severity of* H. pylori* infection.

## 4. Discussion

Epidemiological studies have shown that* H. pylori* is probably one of the most common bacterial infections throughout the world, involving 30% of the population living in developed countries and up to 80%–90% of the population in developing countries [19].

The treatment of* H. pylori* is difficult, requires a two-week application of at least three medicines (proton pump inhibitors and two antibiotics) simultaneously, proves successful in only 80%–90% of cases, and is connected with the risk of adverse effects of therapy with antibiotics (15%–30% of the treated) [20, 21].

A synergistic interaction between* H. pylori* infection and diet has been suggested [22]. In this research, the dietary factors were evaluated. The aim of this retrospective study was to investigate potential differences in the dietary habits of patients with* H. pylori* infection (group I) and in the control not-infected group (group II). We demonstrated a significantly higher consumption of fruit, vegetables, and vitamin C among persons who were not infected. Vitamin C is highly concentrated in stomach mucosa and gastric juice and probably lowers the risk of gastric cancer and influences the course of* H. pylori* infection through a number of mechanisms [23, 24]. It has a positive impact on the stimulation and activity of granulocytes, macrophages, and lymphocytes and the production of immunoglobulin. The direct inhibitory impact of this vitamin on the growth of* H. pylori* is now being examined. Jarosz et al. [13] showed that four-week treatment of* H. pylori* infected patients with chronic gastritis with a high dose of vitamin C caused* H. pylori* eradication in 30% of cases. In those patients, a highly significant rise in gastric juice total vitamin C concentration was demonstrated, which persisted for at least four weeks after treatment. Ruiz et al. [25] found a causal association between* H. pylori* infection and low ascorbic acid levels in the gastric juice. Their findings supported two hypotheses that explain this phenomenon: increased oxidation and decreased secretion of ascorbic acid.

Also diet may provide noxious agents that contribute to* H. pylori* pathogenicity or protective agents that hamper its activity as related to the appearance of cancer diseases. Production of nitrosylating species plays a fundamental role in this regard. Observational cohort studies should ideally provide much more reliable evidence. Analysis of the data substantially confirmed the significant increased risk of developing GC due to high intake of total carbohydrates, processed meat, refined grains, and saturated fat [26, 27]. Prospective Investigation into Cancer and Nutrition study reported a significant increase of noncardiac cancer risk associated with intake of total meat, red meat, and processed meat [28].

Previous studies have reported the protection role of allium vegetables (onion) in gastric cancer [27–29]. In this study, we also observed negative significant relation between onion consumption and* H. pylori* infection.

In this study there was positive relation between carbohydrate, sausages (*P* = 0.001), hamburgers (*P* = 0.002), fat mayonnaise (*P* = 0.002), and soft drinks intake (*P* = 0.001) with* H. pylori* infection.

A high salt concentration in the stomach destroys the mucosal barrier, favors colonization by* H. pylori*, and leads to inflammation and damage-causing gastritis and diffuse erosion [30]. Processed meats have high salt concentration that contributes to* H. pylori* pathogenicity.

We observed a significant difference in intake of unsaturated fatty acid between two groups. It is thought that unsaturated fatty acid may inhibit gastric carcinogenesis. This inhibitory role was experimentally shown in mice [31]. An inverse association was reported between consumption of unsaturated fat and* H. pylori* with a significant dose dependency [32].

Although information on a potentially protective effect of vegetable oil consumption (unsaturated fat) or regarding specific types of unsaturated fatty acids and gastric cancer is very limited, but, in some studies, protective effect of vegetable oil consumption in gastric cancer has been reported [33].

Some studies of healthy adults demonstrated subclinical deficiency of some B vitamins and high prevalence of* H. pylori* infection [34].

*H. pylori* stimulates the macrophage system through the l-arginine/nitric oxide (NO) pathway [35]. As a consequence, chronic* H. pylori* infection of the human stomach may increase endogenous NO formation, yielding, after oxidation, the nitrosating agents N_2_O_3_ and N_2_O_4_, which can produce nitrosamines or cause other types of DNA damage [36]. Nitrosating organisms are capable of catalysing a reaction between nitrite and other organic nitrogen compounds present in the gastric juice to form potent genotoxic N-nitroso compounds (NOC) [37]. Nitrite levels in the gastric juice are increased during hypochlorhydria, which typically occurs during* H. pylori*-induced atrophic gastritis. This may result from bacteria within the stomach generating nitrite from dietary nitrate [38].

In present study among the micronutrients reported, there were significant differences in intake of folate between the healthy subjects and patient groups.

It has been established that chronic* H. pylori* infection causes atrophic gastritis and decreased absorption of vitamin folic acid in patients with this condition. Because methylation of homocysteine to methionine requires folate, an increased plasma homocysteine level is found in* H. pylori*-infected patients [14]. Our study has several potential limitations. First, we did not collect information about lifestyle. Second, our results could also be affected by measurement error in dietary intake, a common limitation of cross-sectional studies. However, prospective cohorts study is required, which evaluates the interaction between dietary habits and* H. pylori* infection. Third limitation was that level of education, socioeconomic, and race of subjects are not considered.

## 5. Conclusion

In summary, the present study on food interaction and* H. pylori*-infection displayed that a diet rich in fruit and vegetables and poor in meat, fat, and salt has a good prophylactic potential for cancer. Fruits and vegetables are sources (among other protective agents) of ascorbic acid, which were associated with a high effect against the progression of gastric carcinogenesis.

Thus, “diet for cancer prevention” can be recommended as a general role of well-being and can represent the basis for a rational health policy.

## Figures and Tables

**Table 1:** Nutrient intakes of healthy group and patient group per day.

Dietary factors	Healthy subjects group Mean ± SD	Patient group Mean ± SD	Pv
Calories (kcal/d)	**2110** ± **514**	**2491** ± **415**	**0.01**
Carbohydrate (g/d)	**300.01** ± **101.4**	**384.9** ± **109.02**	**0.02**
Protein (g/d)	79.6 ± 21.3	81.4 ± 27.3	0.837
Fat (g/d)	65.4 ± 28.9	96.6 ± 31.5	0.279
PUFA (g/d)	**24.31 ± 12.9**	**10.51 ± 11.7**	**0.02**

**Table 2:** Food frequency data on meat samples from healthy group and patient group (per a week).

Meat	Healthy subjects group mean (minimum and maximum)	Patient group mean (minimum and maximum)	*P*
Mutton	1.25 (0.02, 4.7)	1.27 (0.01, 5)	0.714
Veal	2.23 (0.27, 7)	2.1 (0.2, 8.1)	0.317
Chicken	3 (0.26, 7.1)	3 (0.23, 7)	0.894
Fish	**3 (0.47, 7)**	**1 (0.02, 4)**	**0.001**
Egg	2 (0.04, 7)	2 (0.07, 8)	0.511
Sausage	**1 (0.04, 3)**	**3 (0.08, 9.2)**	**0.001**
Hamburger	**0.56 (0.02, 3)**	**2 (0.08, 7)**	**0.001**

**Table 3:** Food frequency data of Fruits and vegetables intakes of Healthy group and Patient group (per a week).

Vegetables	Healthy subjects group mean (minimum and maximum)	Patient group mean (minimum and maximum)	Pv
Carrot	2 (6, 0.32)	1 (0.7, 2.2)	0.232
Tomato	**6 (0.12, 21)**	**3 (0.15, 20.8)**	**0.001**
Onion	**7 (0.46, 21)**	**3 (0.23, 8)**	**0.002**
Green pepper	**3 (0.04, 7)**	**1 (0.05, 6.09)**	**0.01**
Apple	**4 (0.46, 10.2)**	**1 (0.11, 3)**	**0.002**
Citrus fruits	**4 (0.1, 9)**	**2 (0.03, 6)**	**0.001**

**Table 4:** Nutrient intakes of healthy group and patient group per day.

Micronutrient	Healthy subjects group	Patient group	Pv
Folate (*μ*g)	**392 ± 170.03**	**302 ± 131**	**0.01**
Vitamin A (mg)	530 ± 491.1	517 ± 483	0.912
Vitamin C (mg)	**88 ± 71.02**	**58 ± 53.9**	**0.02**
Vitamin E (mg)	5.98 ± 1.03	6.74 ± 0.85	0.421
Zn (mg)	4.5 ± 3.9	4.2 ± 3.7	0.781

**Table 5:** Nutrient intakes of subject without infection and patients with different infection.

Food item	Without infection	Mild infection	Moderate infection	Severe infection	Spearman's rho	Pv
Calories (kcal/d)	2110 ± 0.514	2230 ± 371.51	2532 ± 421.83	2711 ± 451.65	0.363	0.04
Carbohydrate (g/d)	300.01 ± 101.14	343.76 ± 97.36	390.3 ± 110.5	417 ± 118.1	0.327	0.037
Tomato^*^	**6 ± 4.64**	**3.26 ± 1.53**	**3.04 ± 1.42**	**2.68 ± 1.26**	−0.358	**0.033**
Onion^*^	**7 ± 4.6**	**3.80 ± 1.292**	**3.54 ± 1.203**	**3.12 ± 1.06**	−0.312	**0.038**
Green pepper^*^	**3 ± 0.88**	**1.62 ± 0.47**	**1.51 ± 0.44**	**1.33 ± 0.39**	−0.371	**0.039**
Apple^*^	**4 ± 2.33**	**±1.5 ± 0.57**	**1.42 ± 0.53**	**1.22 ± 0.46**	−0.311	**0.027**
Citrus fruits^*^	**4 ± 2.133**	**1.90 ± 0.72**	**1.80 ± 0.68**	**1.55 ± 0.58**	−0.382	**0.042**
Pea^*^	**0.2 ± 0.77**	**0.17 ± 0.16**	**0.16 ± 0.1**	**0.13 ± 0.08**	−0.527	**0.003**
Fish^*^	**3 ± 1.93**	**2.55 ± .012**	**2.4 ± 0.128**	**1.95 ± 0.09**	−0.348	**0.001**
Sausage^*^	1 ± 0.38	0.85 ± 0.32	1.65 ± .062	2.3 ± 0.87	0.339	0.011
Hamburgers^*^	0.56 ± 0.37	0.47 ± 0.31	0.91 ± 0.6	1.27 ± 0.83	0.312	0.021
Fat mayonnaise^*^	1 ± 0.51	0.83 ± 0.41	1.61 ± 0.8	2.25 ± 1.12	0.325	0.013
Olive and olive oil^*^	**3 ± 1.02**	**2.49 ± 0.84**	**2.34 ± 0.79**	**1.92 ± 0.65**	−0.351	**0.031**
Soft drink^*^	0.9 ± 0.51	0.74 ± 0.41	1.442 ± 0.81	2.012 ± 1.14	0.321	0.017
Honey^*^	**2 ± 0.88**	**1.64 ± 0.72**	**1.56 ± 0.68**	**1.26 ± 0.55**	−0.329	**0.003**

^*^Per a week.
